# Antileukaemic Cell Proliferation and Cytotoxic Activity of Edible Golden Cordyceps (*Cordyceps militaris*) Extracts

**DOI:** 10.1155/2022/5347718

**Published:** 2022-04-22

**Authors:** Singkome Tima, Tawat Tapingkae, Chaiwat To-anun, Parinn Noireung, Phikul Intaparn, Wantida Chaiyana, Jakkapan Sirithunyalug, Pawaret Panyajai, Natsima Viriyaadhammaa, Wariya Nirachonkul, Lapamas Rueankham, Win Lae Aung, Fah Chueahongthong, Sawitree Chiampanichayakul, Songyot Anuchapreeda

**Affiliations:** ^1^Department of Medical Technology, Faculty of Associated Medical Sciences, Chiang Mai University, Chiang Mai 50200, Thailand; ^2^Cancer Research Unit of Associated Medical Sciences (AMS-CRU), Faculty of Associated Medical Sciences, Chiang Mai University, Chiang Mai 50200, Thailand; ^3^Research Center of Pharmaceutical Nanotechnology, Chiang Mai University, Chiang Mai 50200, Thailand; ^4^Mushroom Research and Development Center, Chiang Mai 50200, Thailand; ^5^Division of Plant Pathology, Department of Entomology and Plant Pathology, Faculty of Agriculture, Chiang Mai University, Chiang Mai 50200, Thailand; ^6^Department of Pharmaceutical Sciences, Faculty of Pharmacy, Chiang Mai University, Chiang Mai 50200, Thailand

## Abstract

Golden cordyceps (*Cordyceps militaris*) is a mushroom of the genus *Cordyceps*. It has been used as a food supplement for both healthy and ill people. In this study, the antileukaemic cell proliferation activities of golden cordyceps extracts were examined and compared with standard cordycepin (CDCP) in EoL-1, U937, and KG-1a cells. Wilms' tumour 1 (WT1) protein was used as a biomarker of leukaemic cell proliferation. The cytotoxicity of the extracts on leukaemic cells was determined using the MTT assay. Their inhibitory effects on WT1 protein expression and cell cycle progression of EoL-1 cells were investigated using Western blotting and flow cytometry, respectively. Induction of KG-1a cell differentiation (using CD11b as a marker) was determined using flow cytometry. The golden cordyceps extracts exhibited cytotoxic effects on leukaemic cells with the highest IC_50_ value of 16.5 ± 3.9 *µ*g/mL, while there was no effect on normal blood cells. The expression levels of WT1 protein in EoL-1 cells were decreased after treatment with the extracts. Moreover, cell cycle progression and cell proliferation were inhibited. The levels of CD11b increased slightly following the treatment. All these findings confirm the antileukaemic proliferation activity of golden cordyceps.

## 1. Introduction

**Figure 1:**
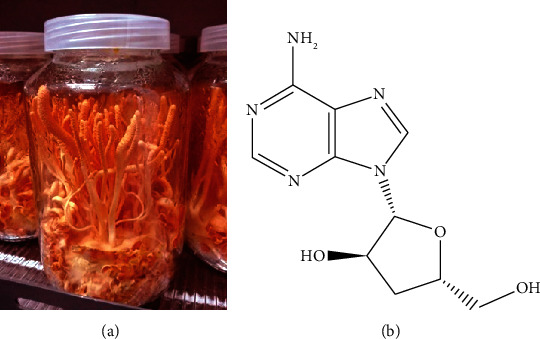
Golden cordyceps. (a) Culture of golden cordyceps (*Cordyceps militaris*) in a sterile bottle. (b) Chemical structure of cordycepin (CDCP).

Phytochemical compounds have been shown to have the ability to control many diseases [1–3]. Cordyceps is one of the medicinal herbs with multipurpose therapeutic benefits. Golden cordyceps (*Cordyceps militaris*) is a bright orange fungus of the genus *Cordyceps* (Figure 1) and belongs to the family Cordycipitaceae [4]. It can infect insect pupae and has effective medical and pharmacological properties. In traditional Chinese medicine, *C. sinensis* (Tibet cordyceps), commonly known as DongChongXiaCao [5], is used in a drug recipe. The active compounds present in cordyceps are cordycepin (CDCP) [6], ergosterol, mannitol, and modified nucleosides that are antiviral and have many pharmacological and medical benefits [7, 8]. Golden cordyceps can grow more easily than *C. sinensis* in the laboratory. The commercial products of golden cordyceps include dietary supplements in the form of capsules or oral liquid cordyceps tonic. In addition, many cordyceps products have been developed for use in traditional Chinese medicine through modern technology as antiaging and cardioprotective agents and for improving sleep, appetite, and immunity. The active ingredients in golden cordyceps are adenosine and CDCP [9]. Moreover, linoleic acid (C18 : 2) and palmitic acid (C16 : 0) are mainly found in the fruiting body and corpus [9]. Xylitol was identified as a novel component from *C. militaris* extract that showed antitumour activity, in addition to the major ingredient, CDCP [10]. A few studies have been conducted to elucidate the antileukaemic activity of golden cordyceps. *C. militaris* ethanol extract has been reported to inhibit tumour growth in the xenograft mouse model bearing murine T cell lymphoma (RMA) through regulation of the p85/AKT-dependent or GSK3*β*-related caspase-3-dependent apoptosis [11]. CDCP (4.4 *µ*g/mL) exerted a synergistic cytotoxic effect with *C. militaris*-mediated apoptosis in human HL-60 leukaemic cells, indicating better a antiproliferative activity of *C. militaris* cultivated fruiting bodies (CM (FB)) than that of cultured mycelia (CM (MY)). This might be due to the significantly higher (approximately 12.5 times) CDCP concentration in CM (FB) than that in CM (MY) [12]. Moreover, CDCP was reported to induce DNA damage, cell cycle arrest, and cell apoptosis, which led to growth inhibition of NB-4 and U937 leukaemic cells [13]. Inhibition of leukaemic cell proliferation is an effective strategy to control the growth of leukaemic cells in patients with noncytotoxic doses of drug treatments. Therefore, it is safe for normal cells. Inhibition of the expression of leukaemia-associated genes or proteins is focused to suppress a target gene expression. Wilms' tumour 1 (WT1) protein has been identified as a biological marker of leukaemic cell proliferation [14]. The WT1 protein level is also associated with leukaemia prognosis [15, 16]. Our previous studies found that several medicinal plants could inhibit WT1 protein expression in various leukaemic cell models. The hexane fractional extract of *Mammea siamensis* (Thai saraphi) flowers inhibited leukaemic cell proliferation via suppression of WT1 protein expression in Molt4 and K562 leukaemic cells [17]. Moreover, curcuminoids, bioactive compounds present in turmeric rhizomes, exhibited a strong inhibitory effect on WT1 protein expression in K562, HL-60, U937, Molt4, and KG-1a leukaemic cells [18, 19]. In this study, the effects of golden cordyceps fractional extracts and standard CDCP on cytotoxicity, WT1 protein expression, cell cycle progression, and cell differentiation in leukaemic cell models were investigated. Moreover, their effects on normal peripheral blood cells, including peripheral blood mononuclear cells (PBMCs) and red blood cells (RBCs), were examined.

## 2. Materials and Methods

### 2.1. Chemicals and Reagents

Standard CDCP, Folin–Ciocalteu reagent, and MTT dye were purchased from Sigma-Aldrich (St. Louis, MO, USA). Roswell Park Memorial Institute (RPMI)-1640, Iscove's modified Dulbecco's medium (IMDM), penicillin-streptomycin, L-glutamine, and trypan blue dye solution were purchased from Gibco (Waltham, MA, USA). Fetal bovine serum (FBS) was purchased from Capricorn Scientific (Hesse, Germany). Ethanol, hexane, ethyl acetate, and dimethyl sulfoxide (DMSO) of analytical grade were purchased from Labscan (Dublin, Ireland). Anti-WT1 antibody (product number OAAN00281) was purchased from Aviva Systems Biology Corporation (San Diego, CA, USA). Rabbit polyclonal anti-GAPDH antibody (product number ABS16) and Luminata™ Forte Western HRP Substrate were purchased from Merck Millipore (Darmstadt, Germany). PE antihuman CD11b (clone ICRF44) was purchased from BioLegend (San Diego, CA, USA). An enhanced chemiluminescence detection kit was purchased from Thermo Fisher Scientific (Waltham, MA, USA). PE anti-CD34 (clone 581) was purchased from BD Biosciences (San Jose, CA, USA).

### 2.2. Golden Cordyceps Material and Extraction

Dried golden cordyceps powder was provided by the Mushroom Research and Development Center (MRDC, Chiang Mai, Thailand). Dried golden cordyceps powder was extracted by maceration using 95% ethanol; the extract was sequentially macerated in hexane, ethyl acetate, and 95% ethanol at room temperature for 3 cycles of 24 h [20]. Crude ethanolic extract (crude EtOH) was obtained after the solvent obtained after maceration in 95% ethanol was filtered through Whatman No. 1 filter paper and removed using a rotary evaporator (Buchi Labortechnik GmbH, Essen, Germany). Hexane fractional extract (Hex), ethyl acetate fractional extract (EtOAc), and ethanol fractional extract (EtOH) were obtained from sequential maceration. Briefly, Hex was obtained after the solvent obtained after maceration in hexane was filtered through Whatman No. 1 filter paper and removed using a rotary evaporator (Buchi Labortechnik GmbH, Essen, Germany). Subsequently, golden cordyceps residue was macerated in ethyl acetate at room temperature for 3 cycles of 24 h. EtOAc was obtained following the solvent obtained after maceration in ethyl acetate was filtered through Whatman No. 1 filter paper and removed using a rotary evaporator. Finally, the golden cordyceps residue was macerated in 95% ethanol at room temperature for 3 cycles of 24 h. EtOH was obtained after the solvent obtained following maceration in 95% ethanol was filtered through Whatman No. 1 filter paper and removed using a rotary evaporator. All extracts were stored at 4°C until further use.

### 2.3. Cell and Cell Culture Condition

EoL-1, U937, and KG-1a leukaemic cell lines were used in this study. The EoL-1 (RBRC-RCB0641) and U937 cells were cultured in the RPMI-1640 medium (Invitrogen™, CA, USA) supplemented with 10% FBS, 1 mM L-glutamine, 100 units/mL penicillin, and 100 *µ*g/mL streptomycin and were incubated at 37°C under 80% relative humidity and 5% CO_2_. The KG-1a was used as the human leukaemic stem cell line model in this study. It was cultured in the IMDM medium (Invitrogen™, CA, USA) supplemented with 20% FBS, 2 mM L-glutamine, 100 units/mL penicillin, and 100 *μ*g/mL streptomycin. All leukaemic cell lines were cultured at 37°C with 5% CO_2_ in a humidified incubator. Anticoagulated blood was collected from healthy volunteers. The PBMCs and RBCs were used for the cytotoxicity assay and RBC haemolysis experiment, respectively. The use of human PBMCs and RBCs in this study was approved by the Human Research Ethics Unit of the Faculty of Associated Medical Sciences, Chiang Mai University (AMSEC-63EM-009).

### 2.4. Cytotoxicity Assay

The cytotoxicity of golden cordyceps extracts was evaluated using the MTT assay. Briefly, the leukaemic cell lines, at a density of 5.0 × 10^4^ cells/well for EoL-1, 1.5 × 10^4^ cells/well for KG-1a, and 1.0 × 10^4^ cells/well for U937, were cultured in 96-well plates containing 100 *μ*L of medium 24 h prior to treatment. Next, 100 *μ*L of fresh medium containing various concentrations (0−100 *μ*g/mL) of the test compounds was added to each well and incubated for 48 h. The MTT dye solution was added (15 *μ*L/100 *μ*L medium), and the plates were incubated at 37°C for 4 h in a humidified 5% CO_2_ atmosphere. Afterwards, 200 *μ*L of DMSO was added to each well and mixed thoroughly to dissolve the dye crystals. Absorbance was measured using a microplate reader (Metertech Accu Reader M965) at 578 nm with a reference wavelength of 630 nm. High optical density readings corresponded to high dye color intensity, representing a high number of viable cells capable of metabolising MTT salt. Fractional absorbance was calculated using the following equation:

(1) $$\begin{array}{c} \text{\% cell viability}=\frac{\text{Mean absorbance in test well}}{\text{Mean absorbance in vehicle control well}}\times \;100. \end{array}$$

The average cell survival obtained from three independent experiments was plotted as a dose-response curve. The 50% inhibitory concentration (IC_50_) of active substances was determined as the lowest concentration that reduced the cell growth by 50% in treated culture compared to untreated or vehicle control culture (0.2% DMSO in culture medium). The IC_50_ values were recorded as the mean ± standard deviation (SD) and compared to determine the cytotoxic activity of active substances. In addition, the cytotoxic effects of the chemotherapeutic drug doxorubicin (Dox), which was used as a positive control for the MTT assay, in all leukaemic cell lines were investigated.

On the other hand, the cytotoxicity of golden cordyceps extracts on normal PBMCs, both of nonproliferating PBMCs and proliferating PBMCs, was also evaluated. PBMCs were collected from three healthy volunteers using the Ficoll-Hypaque density gradient centrifugation method. PBMCs were cultured at a concentration of 1.5 × 10^4^ cells/well and treated with various concentrations of golden cordyceps extracts, following the similar protocol for leukaemic cell lines. According to the strongest antileukaemia activities, EtOH, Hex fractional extracts, and standard CDCP were used to examine their cytotoxic effects on proliferating PBMCs. The PBMCs were incubated with 2% v/v of phytohemagglutinin (PHA) in the RPMI-1640 medium for 24 h. Then, various concentrations of golden cordyceps extracts were added and incubated for another 48 h. The MTT assay was carried out, and the IC_50_ values were compared with those of leukaemic cell lines.

### 2.5. RBC Haemolysis Test

The effect of golden cordyceps extracts on normal RBCs was determined using the RBC haemolysis test. Whole blood was collected from healthy volunteers and a complete blood cell count (CBC) was performed. Five blood samples with normal RBC parameters were included in this study. Fresh whole blood (50 *μ*L) was incubated with 50 *μ*g/mL of golden cordyceps extract at 37°C for 30 min. The treated blood samples were diluted with 1.45 mL of phosphate-buffered saline (PBS, pH 7.4) and centrifuged at 3500 rpm for 5 min. The supernatant was used to measure the absorbance of haemoglobin at 405 nm. The baseline haemolysis sample was treated with DMSO only and diluted with 1.45 mL of PBS (pH 7.4), whereas the complete haemolysis sample was prepared by diluting the treated blood sample with deionised distilled water.

### 2.6. Western Blot Analysis

EoL-1 cells (1.5 × 10^5^ cells/mL) were used as a model for Western blot analysis to evaluate the expression level of WT1 protein after treatment with golden cordyceps extracts. The cells were treated with noncytotoxic doses of extracts and standard CDCP, as determined by the MTT assay, for 48 h. The cells were then harvested; the viable and dead cells were counted after staining with 0.2% trypan blue, and whole proteins were extracted using radio immunoprecipitation assay (RIPA) buffer (50 mM Tris-base, pH 7.4, 0.1% SDS, 1% Triton X-100, 150 mM NaCl, and 1 mM EDTA). Protein concentration was determined using the Folin = Lowry method. Whole protein lysates (30 *μ*g/lane) were separated by 12% SDS-PAGE under reducing conditions and transferred to PVDF membranes. The membranes were subsequently blocked using 5% skim milk in PBS (pH 7.4) at 25°C for 2 h. They were probed with a rabbit polyclonal anti-WT1 antibody and a rabbit polyclonal anti-GAPDH antibody at 1 : 1000 dilution in PBS (pH 7.4) at 25°C for 2 h. Following incubation, the membranes were rinsed six times for 5 min each time with 0.1% Tween-20 (Sigma-Aldrich, MO, USA) in pH 7.4 PBS (PBS-T). Subsequently, HRP-conjugated goat antirabbit IgG (Cat. No. W401B, Promega, WI, USA) was added at 1 : 20000 dilution in PBS (pH 7.4) at 25°C for 2 h. After that, the membranes were rinsed six times for 5 min each time with PBS-T. The proteins were visualised using Luminata^TM^ Forte Western HRP Substrate (Millipore Corporation, MA, USA). Finally, the protein band signals were quantified using a scanning densitometer (Bio-Rad, CA, USA).

### 2.7. Trypan Blue Exclusion Method

Following treatment, leukaemic cells were harvested and washed three times with ice-cold PBS (pH 7.4). Then, the cells were resuspended in PBS (pH 7.4). The cell suspensions were appropriately diluted with PBS (pH 7.4) before mixing with 0.2% trypan blue solution at 1 : 2 dilution for cell counting using a haemocytometer. The viable cells showed a clear cytoplasm because trypan blue can be excluded by live cells, whereas the dead cells showed a blue cytoplasm. The viable and dead cells were counted, and the total cell number was calculated.

### 2.8. Cell Cycle Analysis

Cell cycle analysis was performed to evaluate nuclear DNA content using flow cytometry with propidium iodide (PI) staining. In this study, EoL-1 cells were treated with various concentrations of the ethanol fractional extract of golden cordyceps (EtOH). The EtOH extract activity was compared with that of standard CDCP. EoL-1 cells at a concentration of 5 × 10^5^ cells/mL were incubated with or without noncytotoxic doses (IC_20_ values) of the extracts for 48 h to evaluate their dose-dependent effect on cell cycle progression. Following treatment, the cells were washed twice with ice-cold PBS. A single-cell suspension was prepared, and ice-cold 70% ethanol was added to fix the cells on ice for 30 min. The fixed cells were harvested by centrifugation at 1500 rpm for 5 min. The cell pellets were washed with ice-cold PBS and stained with PI solution (0.1% Triton X-100, 8 *μ*g/mL RNase A, 2 mM EDTA, and 20 *μ*g/mL PI) in the dark at 4°C. Subsequently, red fluorescence was measured using Cytomics™ FC500 (Beckman Coulter). A minimum of 50000 events were recorded per sample. The data were analyzed using the FlowJo Vx software.

### 2.9. Cell Differentiation

The induction of leukaemic cells to differentiate into mature cells is one strategy to treat leukaemia. In this study, the induction effect of golden cordyceps extracts on leukaemic cell differentiation was evaluated by detecting an increase in CD11b expression, a myeloid differentiation antigen, using flow cytometry. KG-1a cells at a concentration of 2.0 × 10^5^ cells/mL were treated with various concentrations (20–60 *μ*g/mL) of EtOH, Hex, and CDCP for 5 days at 37°C in a humidified 5% CO_2_ incubator. The cells were harvested and washed three times with PBS at pH 7.4. The cells were incubated in bovine serum albumin (BSA)-PBS solution (1% BSA, 0.02% NaN_3_, and 10% AB serum in PBS, pH 7.4) for 30 min at 4°C followed by incubation with anti-CD11b-PE antibody for 30 min. The cells incubated with anti-CD34-PE were used as positive controls, whereas the cells incubated with 1% BSA-PBS solution only were used as negative controls. Finally, the cells were washed, fixed with 1% paraformaldehyde in PBS, and 10000 events were analyzed using Cytomics™ FC500. The cell debris was discriminated by gating out of a very small particle population at the lower left corner.

### 2.10. Statistical Analysis

The results are expressed as the mean average ± SD of triplicate experiments. The levels of target protein expression were compared with those of the vehicle control in each experiment. Differences between the mean of each sample were analyzed using one-way analysis of variance (one-way ANOVA). Statistical significance was set at *P* < 0.05 and *P* < 0.001.

## 3. Results and Discussion

### 3.1. Golden Cordyceps Extraction

All golden cordyceps extracts, including crude EtOH, EtOAc, EtOH, and Hex, were semisolid masses with a golden-brown color. Crude EtOH had the highest yield (7.6 ± 2.2% w/w), followed by EtOH (3.6 ± 1.4% w/w), Hex (1.8 ± 0.6% w/w), and EtOAc (0.7 ± 0.3% w/w), respectively (*P* < 0.05). Since ethanol generally extracted polar compounds from natural source and hydrophobic solvents, such as hexane, generally extracted nonpolar compounds, golden cordyceps was found to contain a higher amount of polar compounds than nonpolar compounds because the yield of crude EtOH was significantly higher than that of Hex (*P* < 0.05). On the other hand, EtOH had a significantly lower yield than that of crude EtOH because some nonpolar and semipolar compounds had already been removed by hexane and ethyl acetate. Our previous study identified the presence of many bioactive compounds in golden cordyceps extracts. EtOH extract contained a high content of total phenolic compounds (Folin = Ciocalteu method) and total flavonoids (Aluminum chloride colorimetric method), as well as the main bioactive compound, CDCP. The CDCP content in crude EtOH and EtOH extracts was 1.74% and 1.55% w/w, respectively [20].

### 3.2. Cytotoxicity of Golden Cordyceps Extracts

**Figure 2:**
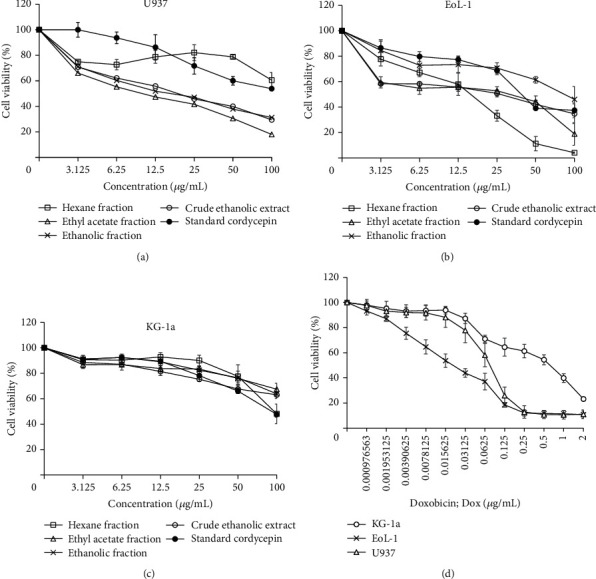
Cytotoxic effects of golden cordyceps extracts on (a) U937, (b) EoL-1, and (c) KG-1a leukaemic cell lines. (d) Doxorubicin (Dox) was used as positive control. Data are presented as the mean ± SD of three independent experiments.

**Figure 3:**
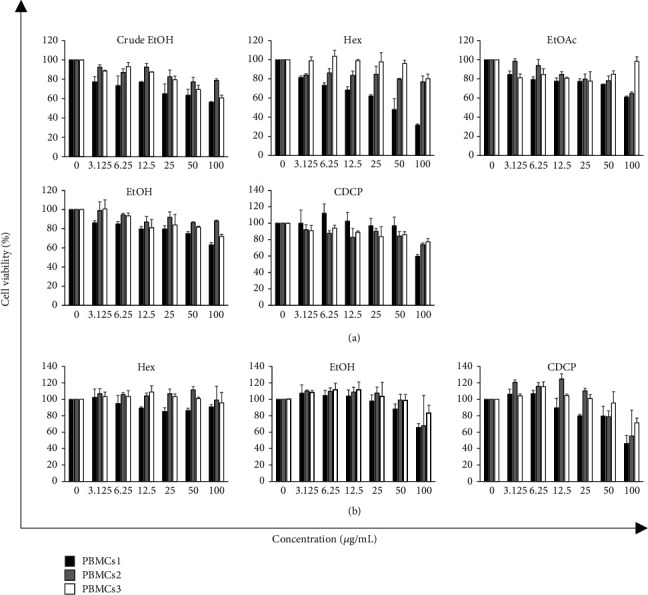
Cytotoxic effects of various golden cordyceps extracts on (a) nonproliferating PBMCs and (b) proliferating PBMCs of three healthy volunteers. Data are presented as the mean ± SD of three independent experiments.

The cytotoxic effects of the extracts from golden cordyceps, including crude EtOH, EtOAc, EtOH, and Hex, were evaluated and compared with those of standard CDCP using the MTT assay. Each golden cordyceps extract showed different cytotoxic effects on leukaemic cells depending on the cell type (Figure 2). EoL-1 and U937 cells were found to be more sensitive to the extracts than KG-1a cells. Hex extract demonstrated the greatest cytotoxic effect on EoL-1 cells with an IC_50_ value of 16.50 ± 3.93 *µ*g/mL, but no effect on U937 and KG-1a cells (IC_50_ > 100 *µ*g/mL). Compared with an antileukaemia drug, doxorubicin (Dox), each golden cordyceps extract showed the lowest cytotoxic effect on all leukaemic cell lines. In addition, the cytotoxic effect of each golden cordyceps extract and CDCP on normal PBMCs was investigated. Most of golden cordyceps extracts demonstrated a noncytotoxic effect on both nonproliferating and proliferating PBMCs with an IC_50_ > 100 *µ*g/mL (Figure 3). However, Hex demonstrated a cytotoxic effect in nonproliferating PBMCs of one healthy volunteer with an IC_50_ value of 49.05 ± 8.37 *µ*g/mL, and CDCP demonstrated a cytotoxic effect in proliferating PBMCs of one healthy volunteer with an IC_50_ value of 94.31 ± 6.24 *µ*g/mL. In this study, we suggested that phenotypic and genotypic variations in an individual person may be involved in response of PBMC against the fractional extracts and CDPC [21].

*C. militaris* has been used as a traditional herb for several decades. The ability to grow in the laboratory makes golden cordyceps more attractive than other cordyceps. Cultivation of *C. militaris* in different laboratories yields different levels of bioactive compounds. In this study, *C. militaris* extracts were evaluated for their activity on the leukaemic cell model. *C. militaris* extracts, including crude EtOH, Hex, EtOAc, and EtOH, and standard CDCP were evaluated for their cytotoxic effects on leukaemic cells and normal PBMCs. U937 and EoL-1 cells were more sensitive to *C. militaris* extracts than KG-1a cells. This might be due to differences in their phenotypes. KG-1a cells represent the leukaemic stem cell (LSC) phenotype by expressing CD34^+^/CD38^−^ on their cell surface [19]. Therefore, KG-1a cells are almost quiescent and inactive and less sensitive to the extracts than U937 and EoL-1 cells, which are dividing cells [22]. Moreover, *C. militaris* extracts showed a higher cytotoxic effect on leukaemic cells than that on PBMCs.

### 3.3. Effect of Golden Cordyceps Extracts on Normal Red Blood Cell (RBC) Haemolysis

To evaluate the effects of golden cordyceps extracts on normal RBCs, fresh whole blood samples were treated with various types of golden cordyceps extracts. RBC haemolysis was measured by analyzing the haemoglobin concentration in the supernatant of treated RBCs. It was found that all golden cordyceps extracts and CDCP demonstrated a minimal effect on RBC haemolysis with a haemolysis rate comparable to that of the baseline haemolysis group, as given in Table 1. Thus, all golden cordyceps extracts did not induce RBC haemolysis in vitro. The antihaemolytic activity of golden cordyceps extracts might be related to the prevention of oxidative damage to the erythrocyte membrane owing to the presence of some antioxidants in the extracts [23]. In our previous study, the EtOH extract exhibited a significantly higher antioxidant activity than that of ascorbic acid and the highest free radical scavenging ability against ABTS [20].

### 3.4. Effects of Golden Cordyceps Extracts on WT1 Protein Expression

**Figure 4:**
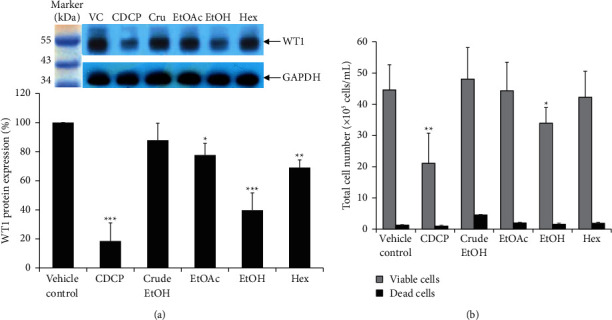
Effects of golden cordyceps extracts on WT1 protein expression in EoL-1 cells. (a) The expression level of WT1 protein (upper band) normalised to GAPDH (lower band). The percentage of WT1 protein expression after treatment with golden cordyceps extracts (crude EtOH, EtOAc, EtOH, and Hex) was compared with that after treatment with CDCP and vehicle control (VC). (b) The total number of viable and dead cells following treatment with golden cordyceps extracts was also evaluated. Data are presented as the mean ± SD of three independent experiments. Asterisks (^*∗*^) denote significant differences compared to the VC: ^*∗*^*P* < 0.05; ^∗∗^*P* < 0.01; ^∗∗∗^*P* < 0.001.

The effect of golden cordyceps extracts on the expression of WT1 protein was evaluated using Western blotting. EoL-1 cells were used as the leukaemic cell model in this study. Cells were treated with noncytotoxic doses (IC_20_ values) of all golden cordyceps extracts and standard CDCP, including 1.5 *µ*g/mL of crude EtOH, 4.3 *µ*g/mL of EtOH, 2.9 *µ*g/mL of crude Hex, 1.5 *µ*g/mL of crude EtOAc, and 8.6 *µ*g/mL of standard CDCP. The cells were harvested and the total protein content was determined. WT1 protein levels were detected and normalised to GAPDH expression. Compared with the vehicle control (VC), the EtOH extract exhibited the strongest inhibitory effect on WT1 protein expression, followed by Hex, EtOAc, and crude EtOH extracts. WT1 protein levels were decreased by 60.2%, 30.9%, 22.4%, and 12.2%, respectively; however, standard CDCP exhibited a greater inhibitory activity (81.5%) on WT1 protein expression (Figure 4(a)). There was no statistically significant difference between the inhibitory effects of the EtOH extract and CDCP. According to our previous study, the EtOH extract contains a high level of CDCP [20], which indicates that CDCP may play a crucial role in WT1 protein expression in EoL-1 cells. In addition, the total number of EoL-1 cells after golden cordyceps treatment decreased in a pattern similar to that of WT1 protein expression, as shown in Figure 4(b). Since WT1 has been reported as a leukaemia marker and plays an important role in leukaemogenesis, various natural products, such as curcuminoids, saraphi flowers, and saraphi seed extracts, have been evaluated for their inhibitory effects on WT1 protein expression [17–19, 24]. This recent finding has confirmed the role of WT1 protein in leukaemic cell proliferation, and golden cordyceps extracts can inhibit WT1 function on cell proliferation induction. In particular, the EtOH extract exhibited an antiproliferative activity in a leukaemic cell model.

### 3.5. Effect of the Ethanol Fractional Extract of *C. militaris* (EtOH) on Cell Cycle Progression

**Figure 5:**
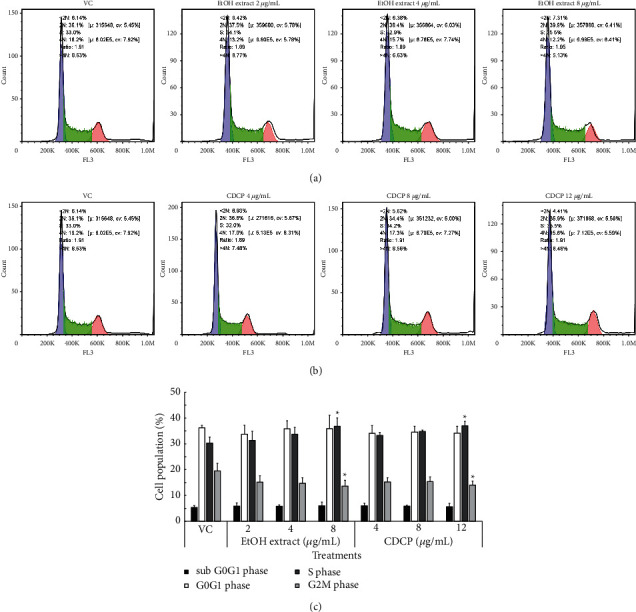
Effect of the ethanol fractional extract of *C. militaris* (EtOH) on EoL-1 cell cycle progression. Cell cycle histograms were obtained after treatment with various concentrations of (a) EtOH fractional extract and (b) standard cordycepin (CDCP). (c) The proportions of EoL-1 cell population in each phase of the cell cycle were analyzed. The data are presented as the mean ± SD of three independent experiments.

The ethanol fractional extract of *C. militaris* (EtOH) was chosen to study cell cycle progression because of its excellent inhibitory effect on WT1 protein expression. EoL-1 cells were treated with various concentrations of the EtOH extract and standard CDCP. The results showed that both EtOH extract and CDCP arrested the cell cycle at the S phase in a dose-dependent manner, with a statistically significant difference compared to CDCP with respect to high doses of the extract (Figures 5(a) and 5(b)). The percentage of the cell population at each phase of the cell cycle is shown in Figure 5(c). Both EtOH extract and CDCP exhibited the same pattern of cell cycle arrest. This result further suggests that CDCP plays a key role in the cell cycle progression of EoL-1 cells. This finding supports a previous study on the effect of CDCP on leukaemic cell progression, which showed that CDCP induced cell cycle arrest at the S phase of the U937 and NB-4 leukaemic cell lines through activation of the Chk2-Cdc25A pathway [13]. In addition, CDCP has been shown to affect the cell cycle arrest and apoptosis in various cancer types such as bladder cancer, gastric cancer, and liver cancer [25–27]. The antitumour and antimetastatic roles of CDCP have also been reviewed [28].

### 3.6. Effects of Golden Cordyceps Extracts on Leukaemic Cell Differentiation

**Figure 6:**
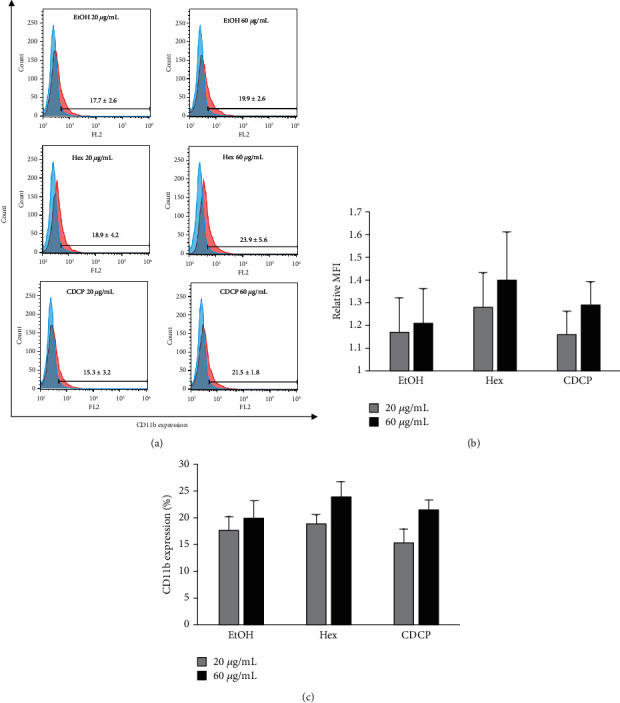
Effects of golden cordyceps extracts and CDCP on KG-1a cell differentiation. (a) Expression levels of CD11b in KG-1a cells after treatment with (red peak) or without (blue peak) golden cordyceps extracts. (b) Relative mean fluorescence intensities (MFI) analyzed by comparing with the vehicle control. (c) The total number of CD11b positive cells measured and compared to that of the negative control group. Data are presented as the mean ± SD of three independent experiments.

The effects of golden cordyceps extracts on the induction of leukaemic cell differentiation were further investigated. The KG-1a cell line was chosen as a leukaemic cell model in this study because of its leukaemic stem cell properties. The effects of the EtOH extract, Hex extract, and CDCP on KG-1a cell differentiation were investigated via CD11b protein expression [29–32]. Following treatment with golden cordyceps extracts, the CD11b positive cell population was slightly increased in a dose-dependent manner, particularly in the Hex extract treatment group. The total number of CD11b positive cells was increased by 15−25% compared with the negative control group (Figure 6). The myeloid differentiation antigen CD11b is a popular marker for evaluating leukaemic cell differentiation [29]. CD11b has been used in many studies as a cell differentiation marker [30–32]. Leukaemic cells can be induced to differentiate using various drugs and small molecules. For example, the treatment of HL-60 leukaemic cells with diperodon and amantadine led to morphological changes in the cells and significantly increased the rate of cell differentiation determined through the increased expression of CD11b [33]. In addition, diallyl disulfide (DADS), an organosulfur compound present in garlic, has been shown to inhibit cell proliferation and induce cell differentiation in HL-60 cells through the increased expression of CD11b [34]. Moreover, the activity of CDCP to reduce the LSC population (CD34^+^/CD38^−^ cells) and exert antileukaemic property has been previously reported [35]. According to the results of this study and all the previous studies mentioned above, golden cordyceps extracts can be used as alternative compounds for the induction of leukaemic cell differentiation.

## 4. Conclusion

In summary, golden cordyceps extracts demonstrated cytotoxicity in various leukaemic cell models without affecting normal PBMCs and RBC haemolysis. Moreover, active compound from golden cordyceps (CDCP) and EtOH fractional extract showed the ability to suppress WT1 protein expression and then decrease total cell number in leukaemic cells. Furthermore, CDCP and fractional extracts (Hex and EtOH) could induce CD11b expression and increase the CD11b cell population in leukaemic cells. Taken together, their inhibitory effects on leukaemic cell proliferation and differentiation suggest their potential of being extensively studied and developed as alternative antileukaemia agents in the future.

## Figures and Tables

**Table 1:** Effects of golden cordyceps extracts on haemolysis of normal RBCs obtained from the blood sample of five healthy volunteers.

Golden cordyceps extracts	% RBC haemolysis (mean ± SD)
Cordycepin	0.81 ± 1.61
Crude ethanolic extract	0.35 ± 0.48
Hexane fractional extract	0.15 ± 0.29
Ethanol fractional extract	0.07 ± 0.10
Ethyl acetate fractional extract	0.88 ± 1.32

Data are presented as the mean ± SD of three independent experiments.

## Data Availability

The data used to support the findings of this study are available from the corresponding author upon request.

## Abbreviations

CDCP: Cordycepin
CD11b: Cluster of differentiation number 11b (cell differentiation marker)
Crude EtOH: Crude ethanolic extract
EoL-1: Human eosinophilic leukaemic cell line
EtOAc: Ethyl acetate fractional extract
EtOH: Ethanol fractional extract
GAPDH: Glyceraldehyde-3-phosphate dehydrogenase
Hex: Hexane fractional extract
KG-1a: Human leukaemic stem cell line
MTT: 3-(4, 5-Dimethylthiazol-2-yl)-2, 5-diphenyltetrazolium bromide
PBMCs: Peripheral blood mononuclear cells
RBCs: Red blood cells
U937: Human monocytic leukaemic cell line
VC: Vehicle control
WT1: Wilm's tumour 1.
